# Structure of junctional epithelium is maintained by cell populations supplied from multiple stem cells

**DOI:** 10.1038/s41598-021-98398-7

**Published:** 2021-09-22

**Authors:** Keisuke Tanaka, Junichi Tanaka, Ryo Aizawa, Mayu Kato-Tanaka, Hiroo Ueno, Kenji Mishima, Matsuo Yamamoto

**Affiliations:** 1 Department of Periodontology, Showa University School of Dentistry, 2-1-1 Kitasenzoku, Ohta-ku, Tokyo, 145-8515 Japan; 2 Division of Pathology, Department of Oral Diagnostic Sciences, Showa University School of Dentistry, 1-5-8 Hatanodai, Shinagawa-ku, Tokyo, 142-8555 Japan; 3 Department of Stem Cell Pathology, Kansai Medical University, 2-5-1 Shin-machi, Hirakata, Osaka 573-1010 Japan

**Keywords:** Periodontitis, Stem-cell differentiation

## Abstract

The junctional epithelium (JE) is an epithelial component that attaches directly to the tooth surface and performs the unique function of protecting against bacterial infections; its destruction causes inflammation of the periodontal tissue and loss of alveolar bone. A recent study that used the single-color lineage tracing method reported that JE is maintained by its stem cells. However, the process by which individual stem cells form the entire JE around a whole tooth remains unclear. Using a 4-color lineage tracing method, we performed a detailed examination of the dynamics of individual stem cells that constitute the entire JE. The multicolor lineage tracing method showed that single-color areas, which were derived from each cell color, replaced all the constituent JE cells 168 d after the administration of tamoxifen. The horizontal section of the first molar showed that the single-color areas in the JE expanded widely. We detected putative stem cells at the external basal layer farthest from the enamel. In this study, JE cells that were supplied from different stem cells were visualized as individual monochromatic regions, and the JE around the first molar was maintained by several JE-specific stem cells. These findings indicated that the JE consisted of several cell populations that were supplied from their multiple stem cells and could help to explore the mechanisms involved in periodontal tissue homeostasis.

## Introduction

The junctional epithelium (JE) is the periodontal tissue that constitutes the gingival epithelium with the oral gingival epithelium (OGE) and the oral sulcular epithelium^1–4^. JE is a non-keratinized stratified squamous epithelium that adheres strongly to the enamel via hemidesmosomes and acts as a barrier against microorganism invasion^5–8^. Owing to the large and highly permeable intercellular space of the JE, gingival crevicular fluid containing antibodies and phagocytes infiltrates the gingival sulcus to eliminate foreign substances^9–14^. Therefore, JE plays an essential role in the innate immune system against the invasion of oral microorganisms from the gingival sulcus and is strongly associated with periodontal disease caused by oral bacterial infections.

The JE turnover in mice takes 4–6 d, a significantly lesser duration than that needed by the OGE (6–12 d)^15–17^. In a previous study that used a bioengineered tooth system, we found that JE cells are derived from the odontogenic epithelium and persist for an extended period of time, about 6 mon^18^, suggesting the presence of JE-specific stem cells distinct from that of OGE-specific stem cells. Using lineage tracing, a more recent report has indicated that Axin2-positive fast-cycling and slow-cycling basal cells contributed to JE maintenance^19^. Proliferative heterogeneity within stem cell populations has been reported in the hard palate epithelium^20^. However, it is unclear whether the clonal proliferation of stem cells can maintain JE homeostasis. Thus, we clarified the clonal dynamics of the JE in this study using the multicolor lineage tracing method.

Multicolor lineage tracing has revealed the clonal dynamics of several organs, such as the brain, intestines, and skin^21–24^. It is noteworthy that leucine-rich repeat-containing G-protein coupled receptor 5 (Lgr5)-positive intestinal stem cells are identified at the bottom of the intestinal crypt using the multicolor lineage tracing method^25–27^. Individual clones of Lgr5-positive stem cells could be easily distinguished by their color using multicolor lineage tracing. Using this method, lingual epithelial Bmi1-positive stem cells were also detected in the oral area. One Bmi1-positive stem cell per interpapillary pit sustained keratinized epithelial cells and was usually in a slow-growing or resting state^28^.

In this study, using the multicolor lineage tracing method, the clonal dynamics that were derived from stem cells in the JE identified.

## Results

### The JE was replaced by a single-color region different from the OGE

**Figure 1:**
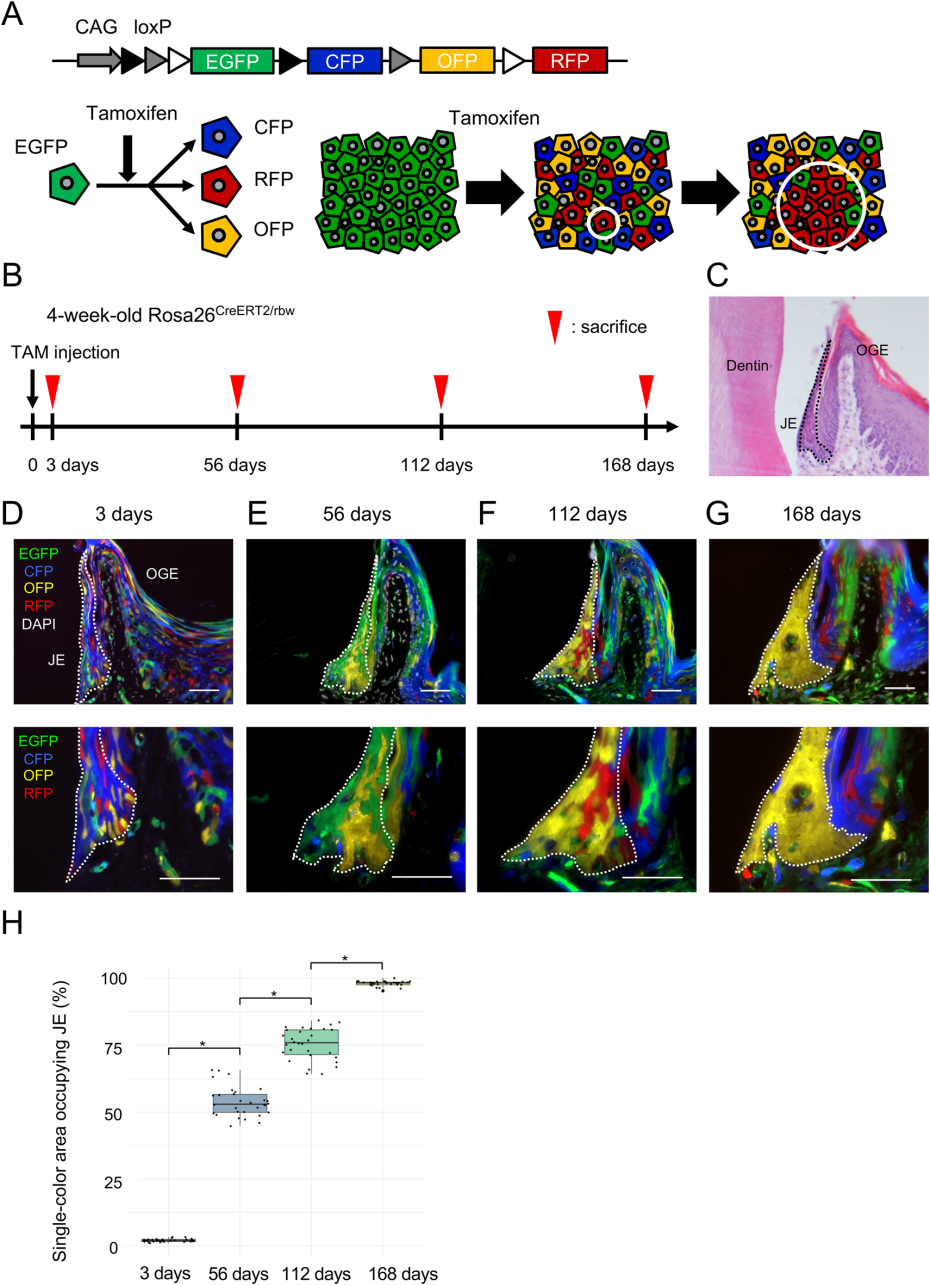
Single-color areas replaced all the constituent cells of the junctional epithelium (JE) over time. (**A**) The strategy to show the presence of stem cells. The upper panel represents a schema of the construct of the Rosa26-rainbow system. The lower panels are a schema of the multicolor lineage tracing method to demonstrate the presence of the stem cells. (**B**) Time course showing the experimental design. (**C**) Representative hematoxylin and eosin (H&E) stained image of the first molar and its surrounding tissue. (**D**, **G**) Representative fluorescence images of the transverse sections of the JE in the Rosa26^CreERT2/rbw^ mice on day 3 (**D**), 56 (**E**), 112 (**F**), 168 (**G**) after tamoxifen administration. The white dotted lines indicate the region of the JE. Representative images were taken from the independent mouse samples (day 3, 56, 112, 168: n = 10 in each group, 5 males and 5 females). (**H**) Quantitative analysis of single-colored areas occupying JE (day 3, 56, 112, 168: n = 10 mice, 30 clones in each group, 5 males and 5 females). The differences between the four groups using one-way analysis of variance, followed by Tukey’s honestly significant difference. Error bars show s.e.m. *P < 0.0001. Scale bars represent 50 μm. *OGE* Oral gingival epithelium, *JE* Junctional epithelium.

We used the multicolor lineage tracing method on the Rosa26^CreERT2/rbw^ mice^29,30^, wherein CreERT2, an inducible mutant of Cre, is ubiquitously expressed. Cre recombinase is expressed via the administration of tamoxifen. The fluorescent genes directly linked to the CAG promoter are exchanged by excising only one set of three types of lox sequences. When Cre acts, it changes green fluorescent protein to mCerulean (CFP), mOrange (OFP), or mCherry (RFP), and all cells are randomly labeled in four colors. In other words, tissue clonality can be visualized using this multicolor lineage tracking method by observing an increase in the same color in the area over time. It is possible to estimate the stem cells that maintain the tissue by visualizing this clonality (Fig. 1A).

To confirm the presence of cell populations that were derived from cells labeled in four colors, the proliferation of single-colored areas was analyzed at each time point. Four-week-old Rosa26^CreERT2/rbw^ mice with erupted teeth were labeled with tamoxifen and analyzed 3, 56, 112, and 168 d after labeling. We performed histological analyses using a fluorescence microscope (Fig. 1B). The anatomical positional relationship between the first molar and its surrounding tissue is shown (Fig. 1C). Three days after tamoxifen administration, JE and OGE were randomly labeled with 4-color fluorescence (Fig. 1D). At 56–112 d, we detected several large single-color areas in the JE (Fig. 1E,F). At 168 d, all the constituent cells of the JE were replaced by single-color areas different from that of OGE (Fig. 1G). The quantitative analysis showed that single-colored areas that occupied the JE significantly increased in size from day 3 to day 168 (Fig. 1H and Data S1). After long-term observation, all the constituent cells of the JE were found to be composed of a cell population derived from a single-colored area. Furthermore, large clones were widely observed in the JE and OGE, and each was composed of individual clones (Fig. 1G). Given that no single-colored area crossed the boundary between the JE and OGE, it was inferred that the JE has its own stem cell population, separate from the OGE.

### Junctional epithelium-specific stem cells were localized at the external basal cell layer

**Figure 2:**
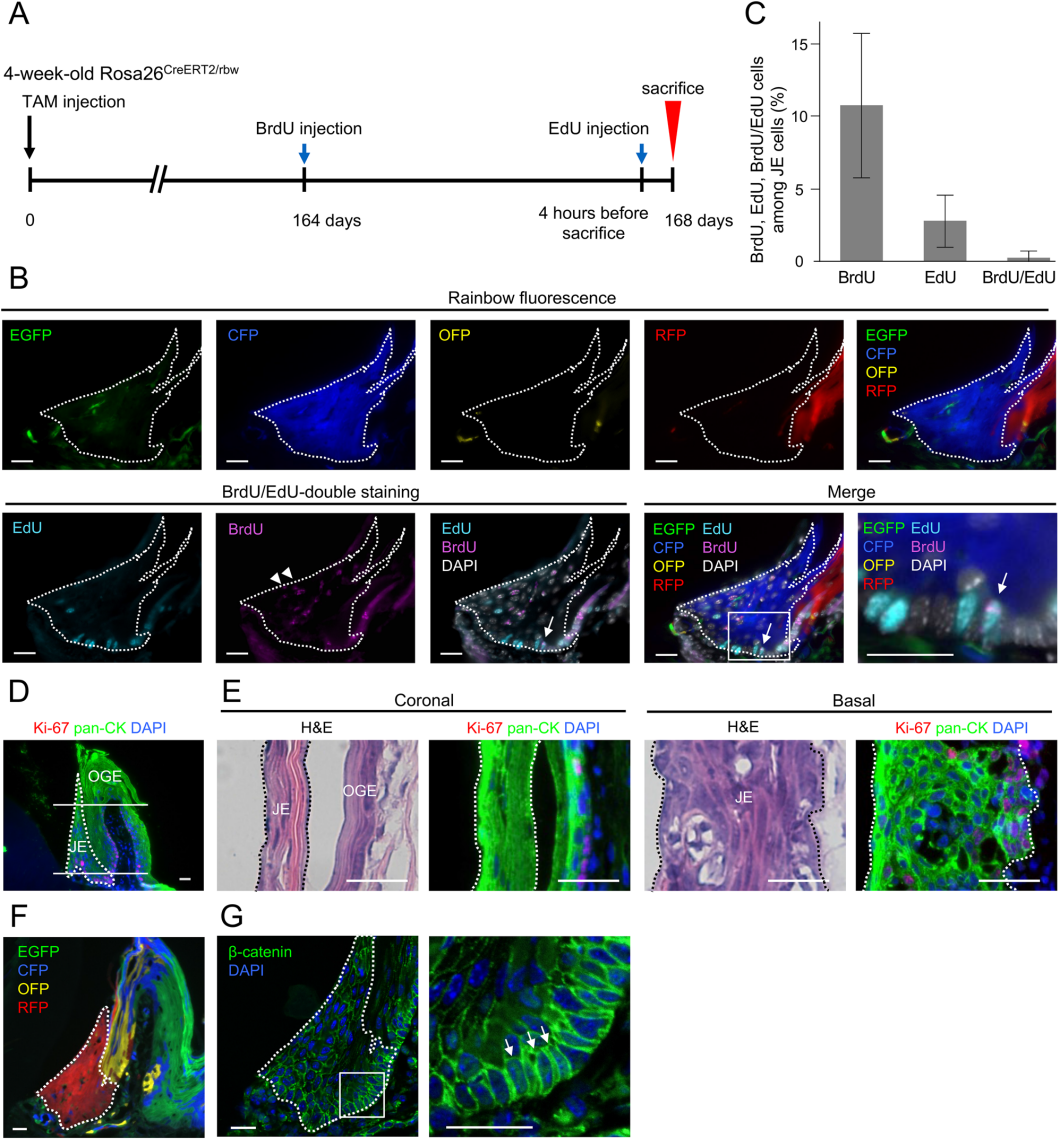
The putative stem cells were located in the external basal cell layer. (**A**) Tamoxifen-labeled Rosa26^CreERT2/rbw^ mice were administered BrdU and EdU 4 d and 4 h, respectively, before euthanasia. The mice were euthanized 168 d after tamoxifen labeling. (**B**) Fluorescent image of the transverse sections of the JE in Rosa26^CreERT2/rbw^ mice 168 d after tamoxifen labeling before BrdU/EdU immunostaining (upper row). Immunofluorescence of BrdU and EdU in the same transverse section (lower left). Merged image of lineage tracing and BrdU/EdU-double staining of the same section (lower right). The enlarged image is presented in the right panel (lower right end). Arrowheads indicate the BrdU label-retaining cells observed on the coronal side. The arrow indicates the BrdU/EdU-double labeled cells in the JE. (**C**) Quantification for the percentage of BrdU, EdU, BrdU/EdU-labeled cells among 1000 cells in JE regions (n = 12 mice, 6 males and 6 females). (**D**, **E**) Immunofluorescence of Pan-CK and ki67. The upper first molars of the WT mice were analyzed horizontally. (**D**) Histological images of the transverse sections. The upper white line sliced the coronal side horizontally, and the lower white line sliced the basal layer horizontally. (**E**) Hematoxylin and eosin (H&E) stained and Pan-CK + ki67 immunofluorescence images of the horizontal sections of the WT mice. The left panel is the coronal side, and the right panel is the basal layer. The dotted lines indicate the region of the JE (n = 3, 2 males and 1 females). The scale bars represent 20 μm. (F) Fluorescence image of the transverse sections of the JE in Rosa26^CreERT2/rbw^ mice 168 d after labeling with tamoxifen. (**G**) Immunofluorescence of beta-catenin in the same transverse section as (**F**). The enlarged image is in the right panel. The arrow indicates the nuclear colocalization of beta-catenin in the JE (n = 3, 2 males and 1 females). The white dotted lines indicate the region of the JE occupied by single-color clones.

Tamoxifen-labeled Rosa26^CreERT2/rbw^ mice were administered bromodeoxyuridine/5-bromo-2′-deoxyuridine (BrdU) 4 d before euthanasia and 5-ethynyl-2′-deoxyuridine (EdU) 4 h before euthanasia to identify the label-retaining cells in S-phase that existed in the JE. The mice were euthanized 168 d after tamoxifen labeling (Fig. 2A). The EdU-labeled cells were located in the basal cell layer in the JE. Although most of the BrdU-labeled cells appeared to migrate to the coronal side (white arrowhead), few cells showing double positivity for BrdU/EdU were detected at the farthest point from the tooth surface of the external basal cell layer on the single-color area of the JE (white arrow) (Fig. 2B, C). The upper first molars of the WT mice were analyzed horizontally. In JE, Ki67, a marker of proliferating cells, was not observed on the coronal side and was only noted in the basal cell layer (Fig. 2D,E).

Recent trials have shown that Wnt-responsive stem cells contribute to the self-renewal of the JE^19^. In agreement with this, the immunofluorescence analysis revealed that the nuclear colocalization of beta-catenin was located at the external basal cell layer in the single-colored area (white arrow) (Fig. 2F,G). These results, which were based on previous reports^19^, suggested that the JE stem cells were in the external basal cell layer in the JE.

### Proliferation of single-colored areas that were derived from stem cells in the JE around the tooth

**Figure 3:**
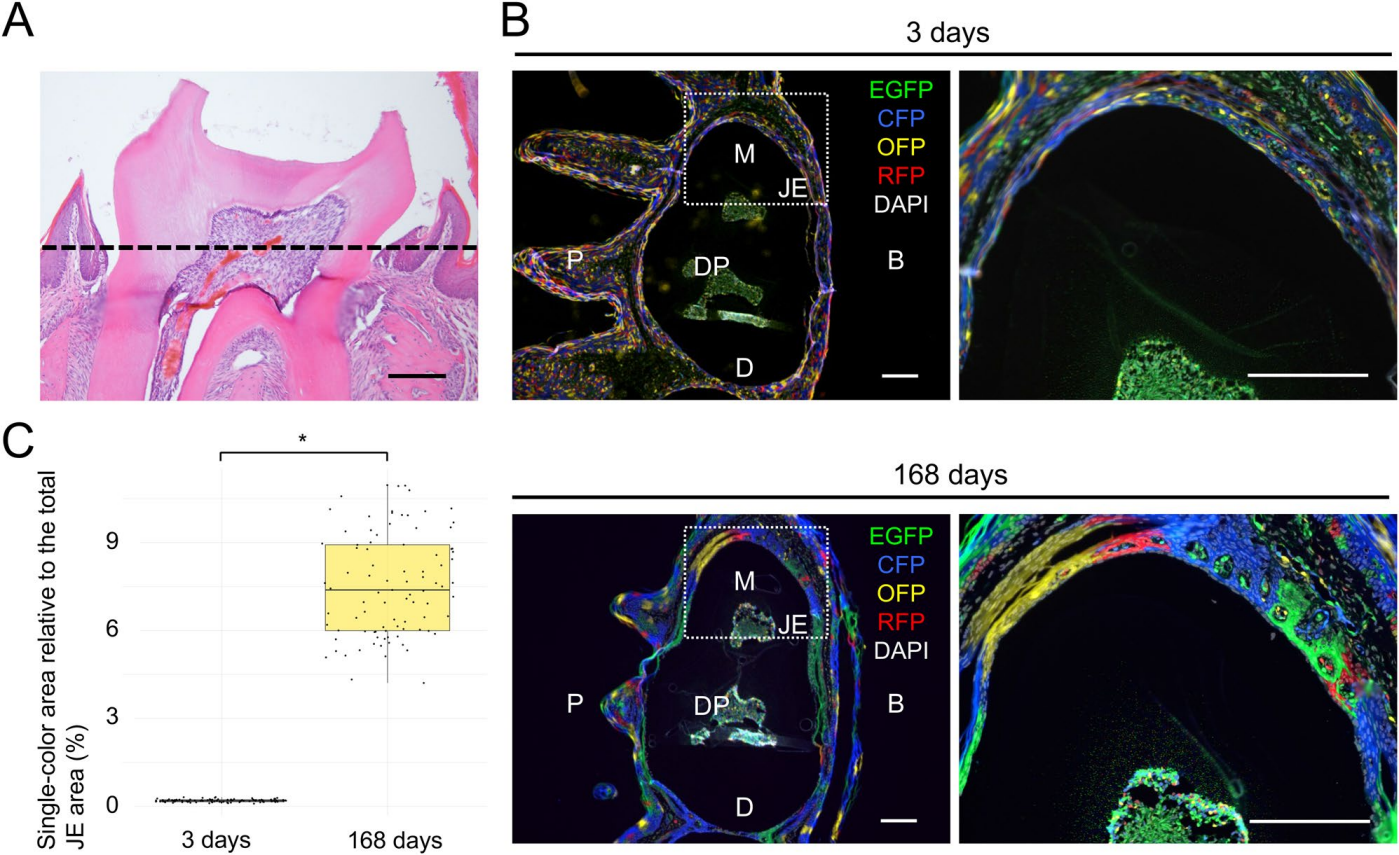
Clonal proliferation of single-colored areas in the JE around the tooth. (**A**) Histological images showing the horizontal sections. The upper first molars were sliced horizontally along the dotted line. (**B**) Representative fluorescence images of the horizontal JE sections in Rosa26^CreERT2/rbw^ mice on day 3 (upper row) and 168 (lower row) after labeling. The enlarged image is shown in the right panel. Representative images were taken from independent mouse samples (day 3, 168: n = 10 in each group, 5 males and 5 females). (**C**) Quantitative analysis of the single-color area relative to the total JE area (day 3, 168: n = 10 mice, 91 clones in each group, 5 males and 5 females). The statistical significance in the differences between two groups was determined using Student’s *t* test. Error bars show s.e.m. *P < 0.0001. The scale bars represent 200 μm. *M* Mesial, *D* Distal, *B* Buccal, *P* Palatal, *DP* Dental pulp.

To examine the distribution of single-colored areas derived from a stem cell over the entire circumference of the first molar, we sliced the upper first molars of the tamoxifen-injected Rosa26^CreERT2/rbw^ mice horizontally and observed the entire image of the JE (Fig. 3A). The JE cells with random 4-color fluorescence were observed on 3 d after labeling, while several single-colored areas of different colors were observed 168 d after labeling (Fig. 3B). These results suggested that the JE around the first molar was maintained by region-specific stem cells. The quantitative analysis indicated that 7.54% was the average size of a single-colored patch that occupied the total area of the JE (Fig. 3C and Data S2).

## Discussion

In the present study, we analyzed the clonality of JE using the multicolor lineage tracing method. Our results showed that the JE around the first molar comprised multiple single-color areas that were derived from each differently colored cell 168 d after the administration of tamoxifen, indicating that the JE has its own stem cells that supply JE cells. Consistent with this indication, a recent trial showed that fast-cycling stem cells with a high division frequency and slow-cycling stem cells with a low division frequency are responsible for the maintenance of JE homeostasis^19^. In the study, lineage tracing of Axin2-positive cells was performed to show that the progeny cells occupy the JE in about 5 d. Given that both slow-cycling and fast-cycling stem cells divide and supply cells, the JE was considered to be constituted within a short period. In the experiment wherein only the slow-cycling stem cells survived after tissue damage, the entire JE was dominated by stem cell-derived cells after 7 d, a healing reaction after tissue damage that is believed to be the result of slow-cycling stem cells rapidly undergoing cell division to supply cells^19^. In their system, the cells change color from red to green under the control of the Axin2 promoter. The authors’ study is indeed valuable because they demonstrated that Wnt-responsive stem cells maintain the structure and function of the JE. The lineage tracing system employed in our study differed from the authors. The cells in our system were randomly labeled using four different colors (Fig. 1A). The multicolor lineage tracing method used in our study enabled the visualization of multiple single-colored areas that were derived from different stem cells. This method could be suitable for analyzing the distribution of individual stem cells. However, it is noteworthy that if another stem cell was labeled with the same color and observed on the section, it is difficult to distinguish whether the cluster has a single stem cell origin. In our study, we analyzed each group (n = 10) and confirmed that the monochromatic region was observed in the JE at 168 d after tamoxifen administration.

In this study, we were able to demonstrate that the JE was maintained from stem cells although we could not identify which stem cells, fast-cycling or slow-cycling, existed.

**Figure 4:**
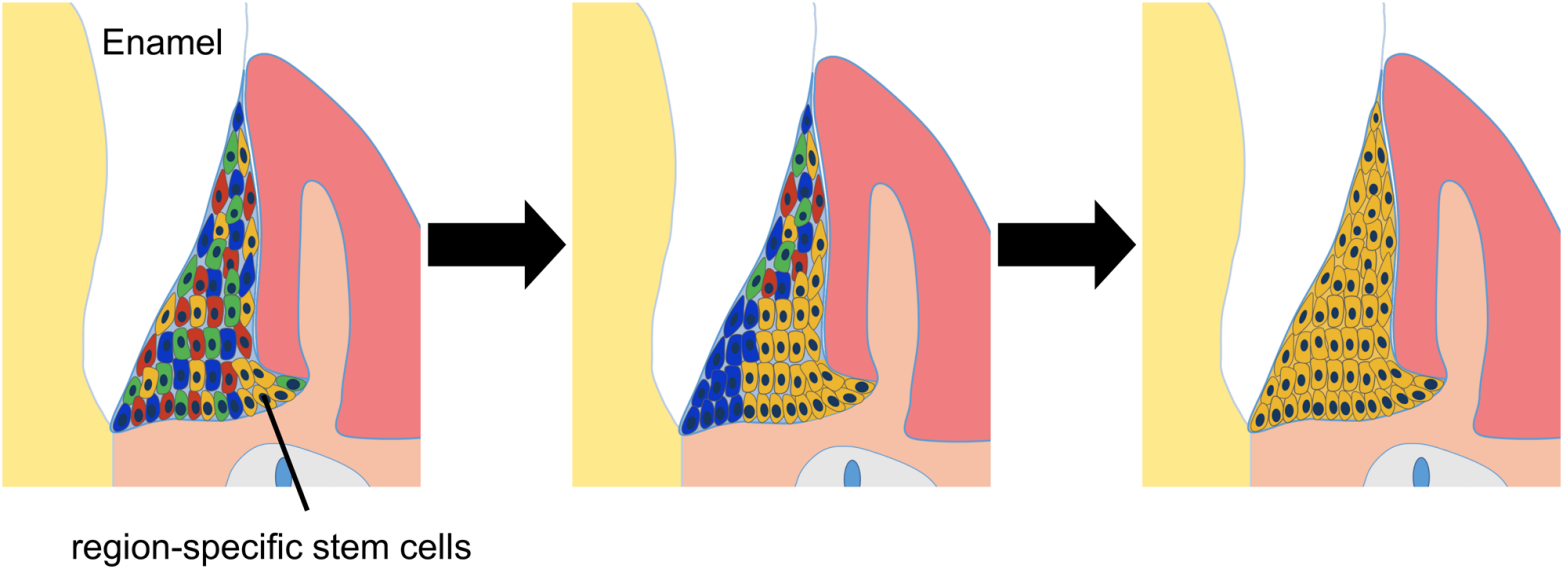
Schematic illustration of the JE composed of single-colored areas suggested by the present results. The JE stem cells were located at the external basal cell layer and supplied JE cells. The JE and OGE were derived from individual stem cells. It was suggested that the JE around the first molar comprised multiple single-colored areas and was maintained by region-specific stem cells.

In the coronal section, the single-colored areas extended from the basal layer to the bottom of the gingival sulcus. In the horizontal section, the single-colored area that established the JE expanded horizontally. Therefore, the JE around the first molar was maintained from multiple single-colored areas. This could mean that a stem cell exists that maintains the structure of JE in each single-colored area. The JE structure could be maintained coronally and horizontally by cell populations that are supplied from multiple stem cells. Figure 4 shows the schematic clonal expansion.

Whether JE cells are supplied by their own stem cell or the OGE-specific stem cells has been a topic of debate for some time. It has been reported that the JE is a part of the OGE component and is maintained by the OGE stem cells^31^. It is widely accepted that during tooth eruption, the reduced enamel epithelium fuses with the OGE to form the primary junction epithelium^32,33^. However, the reduced enamel epithelium is gradually replaced by the OGE.

The JE is non-keratinized, has large intercellular spaces, and adheres to the tooth surface through hemidesmosomes; therefore, the JE differs from the OGE in terms of histology and morphology^34^. Using a bioengineered tooth technique, we demonstrated that the JE of the transplanted teeth is derived from an odontogenic epithelium different from the OGE^35^. However, it is questionable whether the JE can be maintained by its own turnover. We showed that the JE and the OGE were derived from different stem cells, and we successfully visualized their borders. In our previous studies, the JE cells in transplanted tooth germs were replaced by the OGE cells from the basal cell layer over time, and all the JE cells were replaced by the OGE cells 90–150 d after tooth eruption^18^. Previous studies have attempted to verify the process by which the JE derived from odontogenic epithelium replaces cells derived from the OGE. Cell lineage analysis of JE using natural teeth in this study cannot be compared because the purpose of observation was different.

In conclusion, we visualized multiple single-colored areas derived from different stem cells using multicolor lineage tracing method and clarified the distribution of individual stem cells. Our results could provide a basis to understand the mechanisms that are involved in the homeostasis of periodontal tissue.

## Materials and methods

The authors confirm that all experiments were carried out in accordance with relevant guidelines and regulations. The experimental protocol was approved by the institutional review board of Showa University Animal Care and Use Committee (number 12007).

### Animals

Rosa26^CreERT2/+^ and Rosa26^rbw/+^ mice were provided by I. L. Weissman (Stanford University School of Medicine, USA), and C57BL/6 N (wild type: WT) mice were purchased from Sankyo Labo service, Inc. (Tokyo, Japan). All the mice were born and maintained under specific pathogen-free conditions. The rooms in the facility were maintained at 22–26 °C temperature and 45% to 55% humidity, with 60 air changes/h and a cycle of 12 h of light and 12 h of dark. The mice were housed with food and water provided ad libitum. Euthanasia by CO_2_ followed by cervical dislocation was performed and is an accepted method consistent with AVMA Guidelines for the Euthanasia of Animals to minimize the pain or discomfort in animals. This study was carried out according to the Animal Research: Reporting of In Vivo Experiments (ARRIVE) guidelines and the Showa University Animal Care and Use Committee (number 12007).

### Tamoxifen induction

To induce CreERT2-mediated multicolor labeling in all the somatic cells, the Rosa26^CreERT2/rbw^ mice were intraperitoneally injected 4 wk after birth with tamoxifen (Sigma) dissolved in corn oil (Sigma) at a concentration of 9 mg per 40 g of body weight.

### Histological analyses

After euthanasia via cervical dislocation, the maxillae were collected and fixed overnight at 4 °C with 4% paraformaldehyde and decalcified with 10% ethylenediaminetetraacetic acid (EDTA) for 4 wk at 4 °C. The specimens were embedded in an optimal cutting temperature compound (Sakura) and then immediately snap-frozen in liquid nitrogen-cooled isopentane. Thereafter, the frozen sections were cut into 4-μm slices in the coronal or horizontal directions. After three washes with Tris-buffered saline (TBS), the slides were immediately coverslipped with VECTASHIELD Mounting Medium (Vector Laboratories) with 4',6-diamidino-2-phenylindole (DAPI; 1:500 dilution; Dojindo); we examined upper first molars and photographed all the specimens (BZ-9000 fluorescence microscope, Keyence).

### Dual labeling with BrdU and EdU

The Rosa26^CreERT2/rbw^ mice were injected with bromodeoxyuridine/5-bromo-2′-deoxyuridine (BrdU, 30 μg/g) 164 days after tamoxifen administration. Then, the mice were injected with 5-ethynyl-2′-deoxyuridine (EdU, 10 μg/g) 4 days later, and 4 h later they were euthanized. After histological analysis of the upper first molars of Rosa26^CreERT2/rbw^ mice, we removed the fluorescent label and performed double immunofluorescent staining with BrdU and EdU on the same section as per published methods^36^; in brief, we examined the upper first molars and photographed (BZ-9000 fluorescence microscope, Keyence) them. We then treated the sections with 2 N hydrochloric acid to denature the DNA, followed by neutralization with sodium tetraborate. We heated the sections in 10 mmol/L citrate buffer (pH 6.0) for 10 min at 98 °C for antigen retrieval. At this point, the fluorescent label on the specimen was removed. After incubation with blocking solution (Dako) for 30 min, the sections were incubated with anti-BrdU mouse immunoglobulin G (IgG) (Roche) at a 1:100 dilution overnight at 4 °C. After three washes with TBS, the sections were incubated with donkey anti-mouse IgG H&L Alexa Fluor® 594 (Abcam) at a 1:1000 dilution. We performed EdU labeling as per the manufacturer’s detection protocol using the Click-iT EdU Alexa Fluor 488 Imaging Kit (Invitrogen). After three washes with TBS, the slides were immediately coverslipped using VECTASHIELD Mounting Medium (Vector Laboratories) with DAPI. We examined and photographed all the specimens using a BZ-9000 fluorescence microscope (Keyence). The rainbow fluorescence image of Rosa26^CreERT2/rbw^ mice and the BrdU/EdU-double-staining image were merged with ImageJ.

### Immunofluorescence

WT mice were used for pan-CK and ki67 staining. The upper first molars were cut into 4-μm-thick tissue sections in the horizontal direction. The section was heated in 10 mmol/L citrate buffer (pH 6.0) for 10 min at 98 °C for antigen retrieval. After incubation with blocking solution (Dako) for 30 min, the sections were incubated with anti-MS-pan-CK and anti-RB-ki67 at a 1:200 dilution for 60 min. After three washes with TBS, the sections were incubated using mouse Fluor 488 and rabbit Fluor 594 at a 1:400 dilution for 30 min. After three washes with TBS, the slides were immediately coverslipped with VECTASHIELD Mounting Medium (Vector Laboratories) using DAPI. We examined and photographed all the specimens with a BZ-9000 fluorescence microscope (Keyence). Further, we performed immunohistochemistry staining on 4-μm-thick tissue sections from the Rosa26^CreERT2/rbw^ mice 168 d after tamoxifen administration as per a streptavidin–biotin protocol using beta-catenin. Sections of mouse upper first molars at 168 d after tamoxifen administration was observed and photographed with a BZ-9000 fluorescence microscope (Keyence). The same section was heated in 10 mmol/L citrate buffer (pH 6.0) for 10 min at 98 °C for antigen retrieval. At this point, the fluorescent label on the specimen was removed. After incubation with blocking solution (Dako) for 30 min, the sections were incubated with anti-beta-catenin mouse IgG (BD) at a 1:100 dilution for 60 min. After three washes with TBS, the sections were incubated with goat anti-mouse IgG (H + L) cross-adsorbed secondary antibody, Biotin-XX (Invitrogen) for 30 min and then washed three times with TBS. We incubated the sections for 30 min with streptavidin, Alexa Fluor 488 Conjugate (Invitrogen) at a 1:200 dilution. After three washes with TBS, the slides were immediately coverslipped with VECTASHIELD Mounting Medium (Vector Laboratories) using DAPI. We examined and photographed all the specimens with a confocal laser microscope (A1 HD25, Nikon).

### Statistical analyses

ImageJ software was used to evaluate the area of the clone that occupies JE of Rosa26^CreERT2/rbw^ mice n = 40 (day 3, 56, 112, 168: n = 10 mice, 30 clones in each group, 5 males and 5 females). In the quantitative analysis of the horizontal sections, Rosa26^CreERT2/rbw^ mice n = 20 (day 3, 168: n = 10 mice, 91 clones in each group, 5 males and 5 females) were analyzed in terms of the clonal area around the upper first molars using the ImageJ software. BrdU, EdU, and BrdU/EdU-labeled cells among 1000 cells in the JE regions (n = 12 mice, 6 males and 6 females) were counted using ImageJ software. All the data are expressed as the mean ± standard error of the mean. The statistical significance in the differences between two groups was determined using Student’s *t* test. We evaluated the differences between the four groups using one-way analysis of variance, followed by Tukey’s honestly significant difference; *P* values < 0.05 were considered to indicate statistical significance. JMP® 15 (SAS Institute Inc.) was used for the statistical analyses.

## Supplementary Information

Supplementary Information 1.

Supplementary Information 2.
